# Editorial: Improving antimicrobial peptides translational potential through peptidomimetics

**DOI:** 10.3389/fmicb.2023.1304997

**Published:** 2023-10-31

**Authors:** Alfredo M. Angeles-Boza, Nuno C. Santos, Marlon H. Cardoso

**Affiliations:** ^1^Department of Chemistry, University of Connecticut, Storrs, CT, United States; ^2^Institute of Materials Science, University of Connecticut, Storrs, CT, United States; ^3^Instituto de Medicina Molecular, Faculdade de Medicina, Universidade de Lisboa, Lisbon, Portugal; ^4^S-Inova Biotech, Programa de Pós-Graduação em Biotecnologia, Universidade Católica Dom Bosco, Campo Grande, Mato Grosso do Sul, Brazil; ^5^Centro de Análises Proteômicas e Bioquímicas, Programa de Pós-Graduação em Ciências Genômicas e Biotecnologia, Universidade Católica de Brasília, Brasília, Brazil; ^6^Instituto de Biociências (INBIO), Universidade Federal de Mato Grosso do Sul, Cidade Universitária, Campo Grande, Mato Grosso do Sul, Brazil

**Keywords:** antimicrobial peptides, peptidomimetics, bacterial resistance, stapling, virulence factors

Microbial infections represent one of the main threats to human health. It is estimated that multidrug resistant (MDR) microorganisms contribute to over 1.2 million deaths annually worldwide, and it is expected to reach approximately 10 million by 2050 (Murray et al., 2022). This scenario is even more alarming considering the drastic decrease in the discovery and development of new effective anti-infective medicines since the 1980s. Currently, most antimicrobial therapies include monotherapies, in which the most effective agents are often used as a last resort for MDR infections treatment. Additionally, combination therapies have also been proposed for antimicrobial agents with different mechanisms of action and, although controversial, positive outcomes have been reported (Magana et al., 2020). Among the many families of compounds with promising antimicrobial activity, antimicrobial peptides (AMPs) should be highlighted, due to their potency and ability to modulate the immune system. Indeed, the interest in AMPs has greatly increased in the last decade. Naturally occurring AMPs have been identified from most living organisms, including animals, plants, bacteria and fungi. In addition, *de novo* and structure-guided strategies have been used for short AMP design. Finally, considering the groundbreaking advances in bioinformatics, computer-guided design of AMPs and chemoinformatics has demonstrated a tremendous potential to identify physicochemical and structural determinants for a selected biological activity, including antibacterial, antibiofilm and antifungal properties, with high selectivity.

As expected, all these design tools have contributed to generate potent AMP candidates that have been fully characterized functionally and structurally. Consequently, thousands of AMP sequences have been deposited on public databases and used as scaffolds for further AMP design studies (Wang et al., 2016). Nevertheless, it is worth noting the discrepancy of AMP sequences deposited on databases and those submitted to preclinical and clinical trials, thus indicating the limitations to translate this class of antimicrobial to the clinic. Among the main obstacles, we can cite AMP's low bioavailability, toxicity to the host, rapid renal clearance, divergence between *in vitro* and *in vivo* assays, and synthesis cost. As a response to these challenges, small peptide-like molecules called peptidomimetics have been designed to mimic AMPs, potentially presenting improved pharmacological properties.

In this Research Topic, we are delighted to have received three original articles and two reviews submitted by world-renowned scientists working on AMPs and peptidomimetics research and development, based in Brazil, China, Poland, Portugal, United Kingdom, and the United States. These articles highlight: (i) findings regarding N-4-methoxyphenyl-3-(4-methoxyphenyl)-propanamide (AMI 82B) and its significant impact on disrupting *Staphylococcus aureus* virulence in the *Galleria mellonella* model, emphasizing its potential clinical relevance (Mishra et al.); (ii) a detailed exploration of gossypol acetate's mechanism of action, focusing on its interaction with the essential cell division protein FtsZ, also discussing its potential as a novel antimicrobial agent against a wide range of bacteria (Du et al.); (iii) findings on modified anoplins with hydrocarbon staples, elucidating how these modifications lead to improved bactericidal properties, and highlighting the implications for future antimicrobial development (Wojciechowska et al.); (iv) an in-depth review of peptidomimetics as a potential game-changer in the fight against MDR pathogens, discussing their advantages over natural peptide-based drugs, with a core focus on their ability to target virulence factors (Martínez et al.); and, (v) the emergence of antibody-antibiotic conjugates (AACs) as a cutting-edge alternative to conventional antibiotics in the face of the growing challenge of antimicrobial resistance, highlighting successful examples under clinical study (Cavaco et al.).

The first research article is titled “*A substituted diphenyl amide based novel scaffold inhibits Staphylococcus aureus virulence in a Galleria mellonella infection model*”, by Mishra et al.. Here, the authors present a panel of substituted diphenyl amide compounds previously found to disrupt bacterial quorum sensing and that were found to promote survival in the *G. mellonella* model when provided therapeutically to treat a Gram-positive bacterial infection from methicillin-resistant *S. aureus* strain MW2.

The second research article brings the concept of “*Gossypol acetate: a natural polyphenol derivative with antimicrobial activities against the essential cell division protein FtsZ*” (Du et al.). In that work, the authors studied the antimicrobial properties of gossypol acetate against both Gram-positive and Gram-negative bacteria strains, and dig up targets of gossypol acetate, using *in vitro* assays, including investigating its effects on functions (GTPase activity and polymerization) of Filamenting temperature sensitive mutant Z (FtsZ) and its interactions with FtsZ, using isothermal titration calorimetry (ITC), and *in vivo* assays, including visualization of cell morphologies and proteins localization using a microscope. The authors found that gossypol acetate can inhibit the growth of Gram-positive and Gram-negative bacteria. Moreover, gossypol acetate affects cell division in bacteria by interfering with the assembly of the cell division FtsZ ring. Finally, biochemical analysis revealed that the GTPase activity of FtsZ was inhibited and polymerization of FtsZ was enhanced *in vitro*, consistent with the blocking of cell division in the bacteria tested.

The third research article reports on the 10-amino acid residue amphipathic peptide anoplin, “*Stapled anoplin as an antibacterial agent*” (Wojciechowska et al.). Stapled derivatives in positions 2 and 6 (anoplin[2-6]), as well as 5 and 9 (anoplin[2-6]), were synthesized. These modifications led to antibacterial activity comparable or better than that of ampicillin and kanamycin. Importantly, the toxicity toward eukaryotic cells and the hemolytic activity remained low, whereas the proteolytic stability was enhanced. In short, this is an excellent demonstration that stapled peptides are an interesting avenue to improve the translational potential of AMPs.

The Research Topic also contains two review articles. Martínez et al. provide an in-depth review of peptidomimetics as a potential game-changer in the fight against multidrug-resistant pathogens, in “*Peptidomimetics as potential anti-virulence drugs against resistant bacterial pathogens*”. The focus of this review is both on the existing targets, namely the pathogens' secretion systems, biofilms, and quorum-sensing systems, as well as the types of peptidomimetics that have been tested.

In the second review article, “*The use of antibody-antibiotic conjugates to fight bacterial infections*”, Cavaco et al. walk us through the exciting use of antibody-antibiotic conjugates. In this work, we get a deep look at the types of conjugation, the techniques used to characterize these macromolecules, and the structural factors that control absorption, distribution, metabolization, and excretion. Moreover, we are taken to a case study, focused on DSTA4637A (THIOMAB™), a compound in clinical trials that has already been shown to be very effective against methicillin-resistant *S. aureus* (MRSA) strains.

We hope that this Research Topic of original research and review articles serves as a platform to understand recent advances in the field of antimicrobial peptidomimetics, inspiring others to contribute to and expand this rapidly evolving field.

## Author contributions

AMA-B: Conceptualization, Formal analysis, Validation, Visualization, Writing—original draft, Writing—review & editing. NCS: Conceptualization, Formal analysis, Validation, Visualization, Writing—original draft, Writing—review & editing. MHC: Conceptualization, Formal analysis, Validation, Visualization, Writing—original draft, Writing—review & editing.
